# Moderate Intensity Aerobic Exercise Potential Favorable Effect Against COVID-19: The Role of Renin-Angiotensin System and Immunomodulatory Effects

**DOI:** 10.3389/fphys.2021.747200

**Published:** 2021-11-15

**Authors:** Hamid Arazi, Akram Falahati, Katsuhiko Suzuki

**Affiliations:** ^1^Department of Exercise Physiology, Faculty of Sport Sciences, University of Guilan, Rasht, Iran; ^2^Faculty of Sport Sciences, Waseda University, Tokorozawa, Japan

**Keywords:** renin angiotensin system, SARS-CoV-2, coronavirus, inflammation, immune protection

## Abstract

The coronavirus disease (COVID-19) pandemic is caused by a novel coronavirus (CoV) named severe acute respiratory syndrome coronavirus 2 (SARS-CoV-2). As the angiotensin converting enzyme 2 (ACE2) is the cellular receptor of SARS-CoV-2, it has a strong interaction with the renin angiotensin system (RAS). Experimental studies have shown that the higher levels of ACE2 or increasing ACE2/ACE1 ratio improve COVID-19 outcomes through lowering inflammation and death. Aerobic moderate intensity physical exercise fights off infections by two mechanisms, the inhibition of ACE/Ang II/AT1-R pathway and the stimulation of ACE2/Ang-(1–7)/MasR axis. Exercise can also activate the anti-inflammatory response so that it can be a potential therapeutic strategy against COVID-19. Here, we summarize and focus the relation among COVID-19, RAS, and immune system and describe the potential effect of aerobic moderate intensity physical exercise against CoV as a useful complementary tool for providing immune protection against SARS-CoV-2 virus infection, which is a novel intervention that requires further investigation.

## Introduction

Severe acute respiratory syndrome coronavirus 2 (SARS-CoV-2) is the virus that leads to coronavirus disease (COVID-19), which is a novel beta-coronavirus pandemic. The angiotensin converting enzyme 2 (ACE2) is the cellular receptor of SARS-CoV-2 (Chen et al., 2020). ACE is a key regulator of the renin-angiotensin system (RAS). Components of RAS include angiotensinogen (ANG), angiotensin I (Ang I), Ang II, renin, and the ACE (Banu et al., 2020). ACE1 and its homolog ACE2 are the two antagonistic enzymes of the RAS, which balance and offset each other (Tipnis et al., 2000). ACE1 is a key hormonal system that transforms Ang I to Ang II, starting with renin mediating the transformation of ANG to Ang I (Rigat et al., 1990), which induces complicated processes including vasoconstriction, inflammation, and fibrosis through the Ang II type 1 receptor (AT1-R) (Gemmati et al., 2020).

**FIGURE 1:**
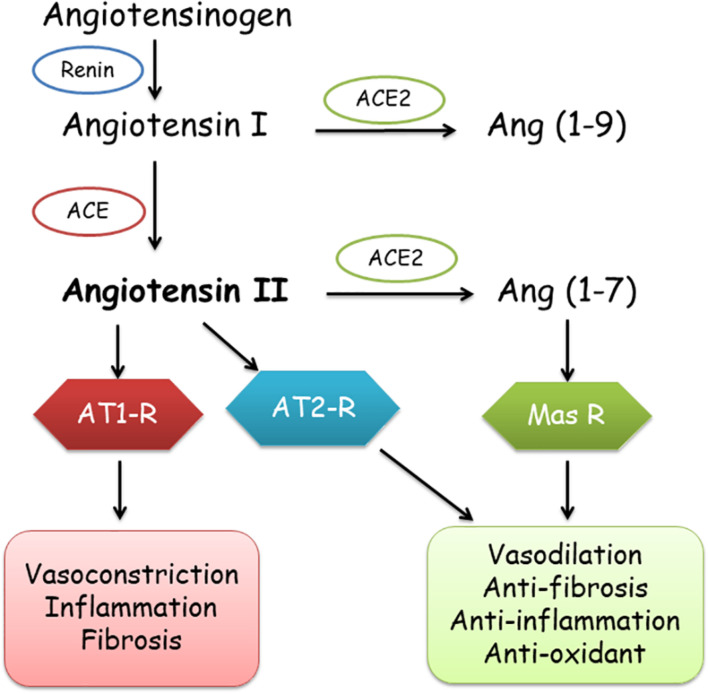
Role of ACE1 and ACE2 in the renin-angiotensin-aldosterone system (RAAS). ACE, ACE2, angiotensin converting enzymes; AT1-R, AT2-R, angiotensin 1 and 2 receptors; Mas-R, Mas receptor.

Contrarily, ACE2 basically leads to a cascade of enzymatic reactions eventuating in the creation of Ang 1–7, by conversion of Ang-II to Ang 1–7 and binding to Mas-related G-protein receptor (Tipnis et al., 2000; Burrell et al., 2004; Ocaranza and Jalil, 2012), which transduces the vasodilator, anticoagulant, anti-inflammatory, anti-fibrosis, and anti-oxidative activities, protecting the organs and blood vessels by counteracting the effects of Ang-II (Gemmati et al., 2020). In addition, angiotensin receptor type 2 (AT2R) also has anti-inflammatory and anti-fibrosis effects (Figure 1).

A negative correlation between ACE2 molecular levels and SARS-CoV-2 fatality has been confirmed (Chen et al., 2020). Physical Exercise (PE) interacts with RAS, one of the most critical pathways in COVID-19, by suppressing or downregulating ACE1 and AT1-R (Evangelista, 2020). As it was stated before, ACE2 is the receptor of SARS-CoV-2. Also PE leads to the higher ACE2, the distinction being that ACE2 pathway provoked by PE increases circulating levels of Ang (1–7) by the activation of ACE2/Ang 1–7/Mas-R axis, which induces an anti-inflammatory and anti-fibrotic effect (Nunes-Silva et al., 2017), but after the initial entry of SARS-CoV-2 through ACE2, consecutive downregulation of ACE2 expression happens, which results in an imbalance between Ang II/Ang 1–7 ratio and exacerbating inflammatory response (Vaduganathan et al., 2020). In fact, SARS-CoV-2 spike protein downregulates ACE2 leading to an overactivated Ang II/AT1R axis, and the detrimental consequences of Ang II may reveal the multiorgan disorders (Banu et al., 2020).

The effects of PE on the RAS are consistent with benefit of RAS inhibitor treatment in COVID-19 infection, which might be potentially economic and effective in lowering the severity of clinical outcome (Evangelista, 2020). Therefore, exercise has been argued as a probable therapeutic strategy against COVID-19 *via* effects on ACE2 (Heffernan and Jae, 2020).

The other critical feature of COVID-19 is the inflammatory response, which occurs in two stages. The first is during the incubation phase of the disease, in which innate immune responses are generated, which can enhance the immune system to resist the virus (Shi et al., 2020). Mild symptoms like fever and cough are probable in this stage. Severe manifestations do not appear in the incubation phase (Zbinden-Foncea et al., 2020).

The second stage of the disease becomes severe in case the patient’s innate immune system is not able to defeat the virus (Shi et al., 2020), and thus the adaptive immune system initiates. Over this second phase, pro-inflammatory cytokines [i.e., interleukin (IL)-2, IL-7, IL-10, granulocyte colony-stimulating factor (G-CSF), interferon gamma-induced protein 10 (IP-10), monocyte chemoattractant protein-1 (MCP-1), macrophage inflammatory proteins-1alpha (MIP-1A), and tumor necrosis factor-alpha (TNF)-α] increase significantly in the blood flow, which is called cytokine storms (Mehta et al., 2020), along with classic symptoms such as fever, cough, fibrosis, and on top of all these, lung inflammation as the major cause of lethal respiratory disorder (Zbinden-Foncea et al., 2020). In individuals with defective immune system, the virus will wipe out concerned cells, especially in tissues with large expression of ACE2 (Zbinden-Foncea et al., 2020).

The progression of COVID-19 symptoms depends heavily on the basic health condition of the person and the immune response provoked by the infection (Shi et al., 2020). The severity of COVID-19 is largely linked with host factors, chiefly cellular immune responses in patients (Zhou and Ye, 2021). Patients with mild COVID-19 and improved patients with severe COVID-19 exhibit a normal immune response to effectively exclude the virus and lastly recover to the preinfection stage (Zhou and Ye, 2021).

A definite adaptive immune response is needed to cease disease progression, and defeat the virus; so interventional approach like exercise to improve anti-inflammatory or constrict pro-inflammatory response should be mainly argued (Zbinden-Foncea et al., 2020). Suppression of IL-1 and IL-6 has been shown to have a therapeutic effect in many inflammatory diseases, including viral infections (Shi et al., 2020). Muscle-derived IL-6 does not act as an inflammatory cytokine, but rather as an anti-inflammatory myokine.

It is safe to exercise during the COVID-19 pandemic for healthy individuals. Active lifestyle boosts the immune function while inactivity suppresses it (Suzuki, 2019). Accordingly, it is of prime importance to stay as active as possible during COVID-19 quarantine period. Some researchers believe that intensity is the only important variable but others have claimed that the influence of training volume, intensity, frequency, exercise selection, exercise variety, sets, reps, rest, and tempo are equally important (Wang et al., 2020).

High intensity exercise (HIE) impairs immune function, which is called “open window” theory; hence, because of originating oxidants and suppression of immune system may be threatening and help to aggravate the COVID-19 disease (Rahmati-Ahmadabad and Hosseini, 2020). Some people infected with the coronavirus (CoV) do not reveal any symptoms for several days; accordingly, HIE should be avoided or applied with caution during the novel coronavirus (2019-nCoV) quarantine period (Rahmati-Ahmadabad and Hosseini, 2020).

It is suggested to use moderate intensity exercise as a non-pharmacological, cheap, and applicable approach to deal with COVID-19 virus (Rahmati-Ahmadabad and Hosseini, 2020).

So, we hypothesized that aerobic moderate intensity exercise may be considered as an adjuvant strategy for increasing ACE2/ACE1 ratio and boosting anti-inflammatory function and as a result decrease severity of COVID-19.

The novel demonstration of the ACE2 enzyme receptor as the main factor of cellular entry by the SARS-CoV-2 and different anti-inflammatory pathways of moderate intensity exercise reveal the priority of investigating the probable modulatory mechanism of these factors in COVID-19 era. Therefore, this paper addresses the hypotheses suggested above expecting that moderate intensity aerobic exercise training approach will add new vision for an improved control of COVID-19 in the general population.

## ACE1 and ACE2 Genes in COVID-19

Significant interindividual variability in susceptibility to severe respiratory infection and disease along with vaccines against infections has been related to extrinsic (socioeconomic status, nutrition, and co-exposures/infections) and intrinsic (age, sex, pre-existing disease, and genetic background) factors (Verhein et al., 2018). Susceptibility of individuals, induced by gene-environment interactions, plays an important role in regulating immunity, survival, and treatment responses in patients from various countries (Gemmati et al., 2020).

ACE1 and ACE2 collaboration in the RAS results in adjustment of the vasoconstrictor/proliferative (ACE1/Ang-II/AT1R) and vasodilator/antiproliferative (ACE2/Ang1–7/MAS-R axis) actions (Gemmati et al., 2020).

Ang-(1–7) has been shown to reverse the actions of Ang II and mediates vasodilation, antitrophic effects, and bradykinin-induced vasodilation (Ocaranza and Jalil, 2012). Increased ACE1/ACE2 ratio and in fact a shift toward ACE/Ang II/AT1R leads to the progression of different complex diseases such as hypertension, atherosclerosis, heart or kidney failure, and severe acute respiratory distress (Gemmati et al., 2020). An interaction between higher ACE2 and Ang-(1–7) and releasing vasoactive factors, such as nitric oxide (NO), prostaglandins, and bradykinin has been suggested, which can reduce the vascular resistance and improve blood flow (Carvalho et al., 2007). Furthermore, a high ACE2/ACE1 ratio preserves vascular endothelial function and exogenous ACE2 activation improves antithrombotic properties as seen in known ACE inhibitor (ACEi) and AT1-receptor blockers (ARB) namely captopril and losartan (Olkowicz et al., 2015). RAS inhibitors increase ACE2 levels to conserve organs from Ang II overload, which keeps the heart across different stresses and may assist patients with COVID-19 with cardiovascular diseases (CVDs) (Ferrario et al., 2020). Rising ACE2/Ang 1–7/Mas-R axis and preventing ACE2 shedding (i.e., ACEis and ARBs) have been presented as potential disease-modifying therapies to minimize greater severity in males (Viveiros et al., 2021).

Prior to the SARS-CoV-2 global pandemic, there were two other lethal CoV outbreaks namely SARS-CoV in 2003 and Middle East respiratory syndrome CoV (MERS-CoV) in 2012. SARS-CoV-2 shares almost 79.5% genomic homology with SARS-CoV and about 50% similarity with MERS-CoV (Lu et al., 2020). The cellular receptor of SARS-CoV-2 and SARS-CoV is ACE2 (Hoffmann et al., 2020; Zhang et al., 2020). Upon binding, viral entry speeds up by the activation of the viral spike (S) protein by a specific transmembrane serine protease 2 (TMPRSS2) that are easily imported in lung tissue (Hoffmann et al., 2020; Zhang et al., 2020).

A decreased AngII/Ang-(1–7) ratio in the renal artery led to lower vasoconstriction and arterial blood pressure (Silva et al., 2015). A high ACE2/ACE1 ratio could explain increased protection against endothelial dysfunctions and vascular disorders, and probably leads to decreased capillary permeability, coagulation, fibrosis, and apoptosis in the alveolar cells, decreasing lung damage sparked by the SARS-CoV-2 (Gemmati et al., 2020). The ACE2 expression in lung facilitates the SARS-CoV-2 entry into lung cells during the infection, so it has a positive correlation with greater risk for evolving the severe form of COVID-19 (Pinto et al., 2020). Viral load is a measure of the amount of virus in an infected person’s given volume of body fluids and is proposed as a way to detect risk of disease severity in COVID-19. In a complete analysis of respiratory tract, plasma, and urine, a correlation between greater prevalence of detectable SARS-CoV-2 plasma viral load and poor respiratory situation and higher inflammation markers (C-reactive protein and IL-6) has been detected (Fajnzylber et al., 2020).

It has been demonstrated that high amount of circulatory ACE2 blocks the SARS-CoV protein binding to its receptor (Vaduganathan et al., 2020). This is in agreement with some studies announcing that ACE2 protects from COVID-19 outbreak (Brojakowska et al., 2020; Chen et al., 2020). The higher primary level of ACE2 in Asian females than in men in line with higher case of fatality in men and lower severity in females suggest a conservative effect of ACE2 on COVID-19 (Chen et al., 2020).

Low prevalence of COVID-19 in premenopausal women in comparison with postmenopausal women or age-matched men, indicates a protective part for estrogen (Wang et al., 2021). The estrogen interacts with the RAS, by prohibiting or downregulating renin, ACE, and AT1-R (Viveiros et al., 2021). Besides, a further X chromosome in females than males is a favorable factor, as several genes associated with immune system are related to X chromosome (Viveiros et al., 2021).

Acute respiratory distress syndrome (ARDS) generated by lower respiratory tract infection leads to a large majority of COVID-19-related deaths (Menter et al., 2020). Patients with Type 2 diabetic (T2D) due to RAS overactivation are more susceptible to serious SARS-CoV-2 infections and ARD (Zhu et al., 2020). Also RAS is overexpressed in obesity (Jia et al., 2020), but it was demonstrated that RAS overactivation in T2D was independent of obesity (Moin et al., 2020).

Obesity indicates an excessive fat accumulation that presents a risk to health especially weak immune system related to serious health conditions (Wang et al., 2020). Approximately, 73% of COVID-19 confirmed cases were overweight or obese with body mass indexes (BMI) over 25 kg/m2 [Intensive Care National Audit and Research Centre (ICNARC), 2020]. There was also a positive relationship between the severity of SARS-CoV-2 and a BMI > 30 (Chiappetta et al., 2020). In a recent study, more than one half (50.8%) of severe COVID-19 cases were obese, classified into four BMI categories, which emphasizes a dose-response link between higher BMI and severe COVID-19 symptoms (Kompaniyets et al., 2021). A meta-analysis showed that obese patients have a higher risk of COVID-19 hospitalization and mortality (Cai et al., 2021).

Angiotensin converting enzyme 2 as a receptor for SARS-CoV-2 is allocated in different human tissues, including adipose tissue (AT). So, a great deal of concentration should be provided on the obese people during the COVID-19 outbreak (Jia et al., 2020).

Besides, the AT incorporates several pro-inflammatory cytokines, which can decline the immune response and, thus, could result the relationship between obesity and the severity of COVID-19 (Al-Benna, 2020; Li, 2020). Obesity increases leptin and decreases adiponectin levels. This inequality causes dysfunction in immune function (Luzi and Radaelli, 2020). Leptin as a cytokine can have pro-inflammatory behavior that affects both innate and adaptive immune responses *via* stimulating the generation of IL-2 and TNF-α and inhibiting the activation of IL-4 and IL-5 (Ali and Sood, 2012). Contrarily, adiponectin is an adipokine that utilizes anti-inflammatory actions that suppresses TNF-α, IL-6, and nuclear factor-κB (NF-κB) and induces IL-10 and IL-1 receptor antagonist (Ali and Sood, 2012).

Regular exercise leads to a negative energy balance and decreases body fat (Gleeson et al., 2011). Regular daily exercise causes training adaptation, which boosts anti-inflammatory activity notably, while along with weight loss (Simpson et al., 2020; Calabrese and Neiman, 2021). The existence of a deleterious microbiota profile in obesity was demonstrated (Quiroga et al., 2020), emphasizing the importance of exercise approach as a potent non-pharmacological strategy in early obesity (Quiroga et al., 2020).

## Physical Exercise and COVID-19

Social distancing has been the initial plan for opposing COVID-19 in many countries (Gasmi et al., 2020). As a result, the world is facing a new challenge, which is the limited use of PE (Filgueira et al., 2021).

The COVID-19 manifestations vary from mild to severe, which depends on the individual’s different metabolic status, provided the age, sex, medical conditions, and lifestyle (Gasmi et al., 2020). Within the several lifestyle factors, exercise deserves a particular consideration. The effect of PE on boosting immune health and preventing infectious diseases (Walsh and Oliver, 2016) and as a useful complementary tool for recovery and prevention against COVID-19 and decreasing its severity (Wang et al., 2020) was suggested. The data suggest that physical inactivity influences the number of infected persons in the older population (Filgueira et al., 2021).

The various health benefits provided by exercise and its safety in asymptomatic people is a good reason to exercise during the CoV outbreak (Zhu, 2020). Experimental studies supported the main idea that PE can cause a shift toward the protecting arm ACE2/Ang 1–7/Mas-R upregulation, whereas concurrently downregulate the ACE/Ang II/AT1-R (Nunes-Silva et al., 2017; Evangelista, 2020).

Exercise has a major effect on susceptibility to infection (Nieman and Wentz, 2019), especially in people susceptible to infectious complications such as autoimmune inflammatory rheumatic diseases (AIIRD), who have immune dysfunction and are at high risk for acute respiratory viral infections and severe COVID-19 (Calabrese and Neiman, 2021). Regular exercise activity has not only protective effects against SARS-CoV-2 infection, but also reduces the severity of infectious events (Grande et al., 2020). Regarding influenza infection, exercise is related to a reduced risk of mortality (Wong et al., 2008). Further, reduced risk of infection and an enhanced immunological response to vaccination are some noticeable clinical outcomes of exercise (Kohut et al., 2004; Weinhold et al., 2016). So the possibility of physical activity to increase the antibody and T cell response to COVID-19 vaccines, specifically in situations experiencing weakened vaccine efficacy like obesity, old age, and inflammation of the joints in arthritis disease (Nieman and Wentz, 2019) and AIIRD (Calabrese and Neiman, 2021), has been proposed. Regular exercise lowers enrollment of leukocytes to AT and consequently lowers the pro-inflammatory state of AT and cause a shift to anti-inflammatory phenotype of many immune cells (macrophages, monocytes, T-cells) (Gleeson et al., 2011).

The interaction between exercise and components of the ACE2/Ang 1–7/Mas-R axis depends on the study design, exercise protocol, intensity, and duration (Nunes-Silva et al., 2017). However, still no data exist in the literature on the exercise mode or intensity to prevent COVID-19.

## Impact of Aerobic Exercise on RAS

Aerobic exercise has been suggested as a potential stressor in the cells of healthy athletes (Nieman, 1994), which cuts down the risk of developing systemic inflammatory processes and stimulates cellular immunity (Nieman and Wentz, 2019).

A shifted RAS balance toward the ACE2/Ang 1–7/Mas receptor axis after aerobic PE was the reason for defensive outcomes facing obesity, insulin resistance, inflammation, high total cholesterol, and triacylglycerol levels (Frantz et al., 2017). Aerobic exercise was proposed as a useful strategy to lower body fat percentage and boosts the skeletal muscle capacity to use oxygen (Booth and Winder, 2005). Continuous aerobic exercise decreases the risk of metabolic disorders (Booth and Winder, 2005; Pedersen and Saltin, 2006). In a study comparing resistance and aerobic exercises, it was shown that the aerobic exercise was more potent in reducing adipose tissue (Slentz et al., 2011). Further, Silva et al. (2017) demonstrated that aerobic PE lowered Ang II and increased Ang 1–7 levels in left ventricular and plasma, adjusted oxidative stress, enhanced antioxidant protection, lowered collagen deposition and inflammatory profile, and reduced blood pressure (Silva et al., 2017).

In a research on mice, the effect of aerobic PE and ACEi treatment on angiotensin pattern was investigated. The exercise training group was placed distinctly in a cage with running wheel (running spontaneously) for 8 weeks. ACEi therapy in combination with aerobic physical activity had a supplementary effect on raising the activity of the ACE2/Ang-(1–7) pathway and in lowering ACE/Ang II activity; suggesting the additive favorable effect of aerobic exercise in ACE-2/ACE ratio (Tyrankiewicz et al., 2021). In the pretraining period, the total ACE was lower in II than deletion/deletion genotype (DD) genotypes, but a similar decrease in total ACE activity in all three genotypes after the aerobic exercise training protocol was demonstrated (Alves et al., 2018).

If aerobic exercise lowers ACE1 activity, it is probable that it may lead to a high ACE2/ACE1 ratio, and as RAS is subject for local tissue homeostasis by anti-inflammatory, anticoagulant, and anti-proliferation, it may control the local trophic responses to viruses (Tikellis and Thomas, 2012), possibly COVID-19. Therefore, aerobic exercise may shift the balance in the RAS toward the potective arm [ACE2/Ang-(1-7) axis] and counterbalance ACE2 repression caused by SARS-COV-2.

## Exercise Intensity and Immune Function

Various physical exercises regarding intensity and type have distinct outcomes on immune system (Nieman and Wentz, 2019). It was demonstrated that unaccustomed strenuous or prolonged exercise may diminish the immune defense function (Zhu, 2020). As such, avoiding long and stressful exercise sessions that one is not used to is recommended (Zhu, 2020). HIE possibly because of originating oxidants and suppression of immune system may be threatening and help to aggravate the COVID-19 virus (Rahmati-Ahmadabad and Hosseini, 2020). In a human study, the acute response of two different exercise regimens of HIIE and moderate intensity continuous exercise (MICE) on plasma and urinary concentration of RAS components was investigated. The HIIE protocol comprised of a 5-min cycling at 60–70% of heart rate peak intensity proceeding by 10 sets of 30 s above 90% with 1 min of recovery and 3 min of cool down. The MICE was performed at a steady power equal to 60–70% of HRp and finished at the same total work of HIE. The results declared that: (1) acute aerobic exercise can balance plasma and urinary levels of ACE and ACE2 in healthy individuals; (2) the MICE protocol led to a higher increase in urinary levels of Ang-(1–7) compared with HIIE (Magalhães et al., 2020). It is probably because HIIE requires a higher demand on the anaerobic metabolism (identified by peak lactate) in comparison with MICE, in which aerobic metabolism is greater (identified by peak non-ester fatty acid) (Cabral-Santos et al., 2015).

An experimental study comparing the effect of different aerobic exercise intensities indicated that moderate intensity aerobic exercise, in comparison with high intensity one, causes a significant rise in fibronectin type III domain-containing protein 5 (FNDC5) and peroxisome proliferator-activated receptor gamma coactivator 1-alpha (PGC-1α) in the muscle tissue of obese Wistar rats (Attarzadeh Hosseini et al., 2021), leading to its antioxidant activity (Wang et al., 2016).

In another study on healthy young men, long-term HIIT [90% maximum heart rate (HRmax), three times a week] but not moderate intensity continuous training (MICT) [70% HRmax, five times a week)], deteriorated immune response by increasing TNF-α (Gerosa-Neto et al., 2016). Additionally, it was indicated that only MICT [not high intensity interval training (HIIT)] favor as a potential type of exercise in SARS-CoV-2 disease, as HIIT cause the repression of the immune system (Rahmati-Ahmadabad and Hosseini, 2020).

On the other hand, some studies have shown the positive effects of HIE on inflammatory factors (Campbell and Turner, 2018; Khaleghzadeh et al., 2020). Campbell and Turner (2018) claimed that acute vigorous exercise does not lead to harmful effect on immune system. In the study by Khaleghzadeh et al. (2020) male obese Wistar rats had done 8 weeks of HIIT, which caused a significant reduction in plasma IL-6, TNF-α, and aspartate aminotransferase (AST). These conclusions are probably because the mentioned studies are either on highly fit subjects (Campbell and Turner, 2018) or on animals (Khaleghzadeh et al., 2020). It seems that having an athletic background might have led to adaptations, which could modify the training effect on immune function. Also, using animal models has limitations, such as species distinction and stress related with enforced exercise, which should be taken into account (Zhu, 2020). Due to the ethics and safety concerns, there are few studies on prolonged HIE in sedentary people (Zhu, 2020).

Endurance training-induced adaptations differ according to the modes of training employed (e.g., running, swimming, cycling), the training length (weeks, months), and the frequency, duration, and intensity of workout (Vellers et al., 2018). While moderate exercise may boost the immune system, exhausting exercise may weaken it. In a study on subjects participating in marathon, a rise in respiratory infection was indicated (Peters and Bateman, 1983), after which J-curve was suggested by Nieman (1994) in the 1990s to define the link between exercise intensity and upper respiratory tract infection (URTI) (Nieman, 1994), indicating that moderate intensity exercise boosts immune function whereas vigorous exercise weakens the immune activity (Walsh and Oliver, 2016). Also, after long vigorous exercise, a phenomenon called “open window” happens for 3 to 72 h, diminishing immunity, resulting in higher incidence of airway infections (Nieman and Pedersen, 1999). Suppression of the immune response caused by vigorous exercise may be due to: (1) elevated number of neutrophils and the concurrent lowered number of lymphocytes in the blood; (2) defective phagocytosis and neutrophil function; (3) reduced release of reactive oxygen species (ROS); (4) reduced natural killer cell cytolytic activity (NKCA); and (5) declined immunoglobulin levels (Moreira et al., 2009). However, it is unclear whether exercise to exhaustion itself or other factors that increase the probability of exhaustion weaken the immune system. Nonetheless, athletes who train very hard typically suffer more frequent infections probably because their high training load increases psychological stress and interrupts their circadian rhythms (Simpson et al., 2020). It is suggested that in competitive sports, upper respiratory symptoms (URS) might be caused by non-infectious causes, which are related to extreme training or competition, for instance mental stress and anxiety, insufficient sleep, travel, dietary deficiency, and climate variations or migration of inflammatory cytokines, originating from the damaged skeletal muscle to the respiratory system (Walsh, 2018).

Besides from athletic populations, regular PE is a key protective factor to improve immune function, while inactivity weakens immunity (Nieman et al., 2011). In a study on 48,440 adult patients with COVID-19, self-reported physical activity level was used to categorize them in three groups (consistently inactive = 0–10 min/week, some activity = 11–149 min/week, and consistently active ≥150 + min/week). Inactive patients with COVID-19 had a higher risk of hospitalization, admission to the intensive care unit (ICU), and death than patients who were constantly active (Sallis et al., 2021). Even doing some physical activity reduced the likelihood of hospitalization, which is suggestive of beneficial effect of physical activity in pandemic period. Being consistently inactive led to higher risk for all outcomes and even exceeded the health risks of smoking and nearly all the chronic diseases (cancer, diabetes, hypertension, renal disease, cardiovascular disease, chronic obstructive pulmonary disease) investigated in this study, arguing physical inactivity as a threat for severe COVID-19 consequences (Sallis et al., 2021). A 12-month observational study on the activity level of 547 healthy adults (age 20–70 years) found that moderate physical activity leads to approximately 20% reduction in URTI risk (Matthews et al., 2002). Physical fitness and moderate intensity training are related to improved types of immune markers and reduce premature mortality rate (Suzuki and Hayashida, 2021). Fit people who perform moderate intensity exercise on a regular basis showed decreased markers of chronic inflammation, the stronger and more durable immune response to vaccination, increased immunosurveillance, and a lower disease susceptibility (Simpson et al., 2020). In a cohort study on 2,690 adults, a significant dose-response relationship between having moderate [adjusted relative risk (aRR) = 0.43, 95% confidence interval (CI): 0.25, 0.75] and high (aRR = 0.37, 95% CI: 0.16, 0.85) cardiorespiratory fitness with lower COVID-19 mortality has been investigated (Christensen et al., 2021). It is suggested that PE can lower the danger, infectious period, and severity of viral infections (Laddu et al., 2021). In addition, the health benefit of an objective of ≥ 150 min per week (an activity that requires moderate effort) was indicated by the American College of Sports Medicine (ACSM) [Bushman and American College of Sports Medicine (ACSM), 2017]. The proposed amount of physical activity in a universal strategy (from 2018 to 2030) by the World Health Organization (WHO) was modest to intense exercise, at least 5 days per week, each lasting 30–60 min [World Health Organization (WHO), 2018], indicating that 150 min/week physical activity duration is the initial amount for keeping health in the normal population.

## Definition of Moderate Intensity Exercise

Present interpretations and definitions of moderate to vigorous intensity exercise are ambiguous. Due to the multiple formats used for illustrating moderate and vigorous intensity exercise in the research literature, presenting intensity to health outcomes is demanding (MacIntosh et al., 2021).

Brisk walking, dancing, and gardening are practical examples for prescribing a moderate intensity activity (MacIntosh et al., 2021).

Heart rate is a prompt assessment of exercise intensity, which should be individualized to target an applicable relative intensity domain (MacIntosh et al., 2021). Heart rate ranges indicated relative to the individual maximal heart rate or percentage of heart rate reserve can be employed to prescribe moderate (40–59% of aerobic capacity reserve or heart rate reserve) and vigorous (60–84% of these reserves) exercise intensity (Warburton et al., 2007).

In case, the individualized testing is not available, rating of perceived exertion (RPE) with a scale of 6–20 can be used in which a mean value of 10.8 ± 1.8 would be at the boundary between moderate and vigorous intensity (Scherr et al., 2013). Another useful method is the talk-test. During exercise, rapid ventilation makes it rather challenging to achieve a conversation. Just below this intensity is considered moderate (Reed and Pipe, 2014).

Although exercising at a percentage of maximal volume of oxygen (consumption) (V̇O_2__max_) and heart rate reserve are surely more applicable than using an absolute speed or power output, or even metabolic equivalent (MET) (MacIntosh et al., 2021), applying a common percentage of those measures probably serves as a diverse degree of disturbance to homeostasis for various individuals (Iannetta et al., 2020). Such as, two individuals exercising at 70% of V̇O_2__max_ may have distinct levels of metabolic disturbance to homeostasis due to exercising in different exercise domains (Jamnick et al., 2020).

Exercise intensity can be given in absolute or relative terms (i.e., relative to body weight, maximal oxygen uptake, maximal heart rate, or heart rate reserve). The ability to maintain homeostasis during acute exercise and the chronic adaptation to training is influenced by genetics, fitness status, comorbidities, and other factors, so, prescription should be individualized based on the expected metabolic disturbance obtained by the exercise (MacIntosh et al., 2021).

## Anti-Inflammatory Signaling Pathways of Moderate Intensity Exercise

It is widely recognized that regular moderate intensity PE boosts the hosts immunocompetence and preserves against infectious diseases (Alack et al., 2019).

Regular moderate intensity aerobic exercise (25–30 min per session/three times a week) for 10 months caused a significant rise in level (titer) of antibodies in the blood after influenza vaccination (Kohut et al., 2004). The effect of moderate intensity exercise on reducing prevalence of acute respiratory infections as well as the common cold, influenza, pneumonia, and COVID-19 has been proposed (Calabrese and Neiman, 2021). Immune response to influenza vaccination has been improved in individuals performing long-term exercise training (Nieman and Wentz, 2019; Valenzuela et al., 2021).

Different molecular pathways about anti-inflammatory effects of PE as a non-pharmacological intervention have been investigated.

One of the crucial parts of the inflammatory mechanism is cytokine originated by the innate macrophages, dendritic cells, natural killer cells, and T and B lymphocytes (Ragab et al., 2020). Moderate aerobic exercise training increases T cell count that is reduced in SARS-CoV-2-infected patients (Li, 2020). Cytokines are classified as pro-inflammatory (e.g., IL-1, IL-6, and TNF-α) and anti-inflammatory (e.g., IL-10 and IL-1 receptor antagonist) (Ragab et al., 2020); the latter can be modified due to physical exercise. Active skeletal muscle develops different cytokines and peptides with anti-inflammatory properties (Alack et al., 2019). Muscle-derived IL-6 does not act as an inflammatory cytokine, but rather as an anti-inflammatory myokine that acts in a hormone-like pattern and eliminates endocrine effects in other organs. IL-6 prompts the generation of anti-inflammatory cytokines such as interleukin-1 receptor antagonist (IL-1ra) and IL-10 (Steensberg et al., 2003), soluble TNF-α receptors (Krüger et al., 2016), and inhibits the endotoxin-induced TNF-α production (Steensberg et al., 2003). A systematic review supported the fact that aerobic exercise advances the immune markers including leukocytes, lymphocytes, lymphocyte subpopulations, ILs, NK cells, and immunoglobulins in healthy individuals (Gonçalves et al., 2020). A destructive inflammatory response with the increased levels of cytokines, a critical life-threating condition known as “cytokine storm,” was detected in patients with severe COVID-19 (Ragab et al., 2020). The overproduction of pro-inflammatory cytokines leads to multiple organ damage if remains uncontrolled (da Silveira et al., 2021). In the lung, alveolar macrophages and epithelial cells are the utmost subject for over-generating of cytokines, which cause alveolar damage due to wall thickening (da Silveira et al., 2021). When SARS-COV-2 causes URTI, it leads to the generation of pro-inflammatory cytokines, including IL-1β and IL- 6, causing mild or severe URTI (Shi et al., 2020). Not only young, but also old active people (>65 years) gain from exercising by an increasing T cell proliferation, NK cell cytotoxicity, and neutrophil phagocytic activity (Walsh et al., 2011).

Regular aerobic exercise reduces the basal concentration of inflammatory cytokines and pro-inflammatory T effector memory CD45+ re-expressing T cells (T-EMRA cells), and inhibits the stimulation of thrombotic pathways (Philippe et al., 2019). Over moderate and intensive aerobic exercise bouts of shorter than 60 min, a significant rise in anti-inflammatory cytokines, neutrophils, NK cells, cytotoxic T cells, and immature B cells occur that have major effect in immune response (Nieman and Wentz, 2019). In a study conducted on two independent groups with cycling as their exercise modality (80% V̇O_2__max_ for 20 min) vs. (+5% of the individual blood lactate threshold for 30 min), the number of peripheral blood B lymphocytes doubled during these acute exercises with a mean increase of 88 and 60%, respectively (Turner et al., 2016). Regular endurance exercise, such as running or walking, has been generally recognized as a way to rise CD4 + /CD8 + ratio in older adults (Walsh et al., 2011).

A study on rats demonstrated that aerobic exercise led to 50% increase in AMP-activated protein kinase (AMPK) activity (Budinger et al., 2008), which attenuates pro-inflammatory cytokines in airway epithelial cells by inhibiting the NF-κB system (Cholewa and Paolone, 2012). Furthermore, AMPK activation leads to ACE2 phosphorylation and increases Ang 1–7 in pulmonary endothelium cells (Zhang et al., 2018). Regular moderate exercise caused the conversion of Ang II to Ang 1–7 and reduced pulmonary fibrosis *via* ACE2/Ang-(1–7)/Mas receptor pathway (Prata et al., 2017). In this regard, exercise may prevent the destructive outcomes of binding SARS-CoV-2 to ACE2 receptor, lowering the pro-inflammatory response in the pulmonary system (Zbinden-Foncea et al., 2020).

Exercise leads to a stimuli of cortisol, principal stress hormone with a potent anti-inflammatory characteristics (Gleeson et al., 2017). Lower cortisol and higher interferon gamma (IFN-γ) levels after exercise (30 min cycling at 115% of their lactate threshold power) led to shifts in NK-cell subsets (Gupta et al., 2018).

Also, there is a positive relationship between the integrity of gut microbiota and local, innate, and systemic immunity (Wiertsema et al., 2021). Microbiota profile was positively modified by exercise intervention (Quiroga et al., 2020). Evidence demonstrates that exercise-induced increase in gut microbiota leads to an improved immune function, which is higher in trained athletes compared with control group (Barton et al., 2018; Mailing et al., 2019).

Exercise reduces the NLR family pyrin domain containing 3 (NLRP3) inflammasome, a key component of the innate immune system that initiates caspase-1 activation, which is an inflammatory form of cell death and the release of pro-inflammatory cytokines IL-1β and IL-18 (Quiroga et al., 2020; Ding and Xu, 2021). It was recommended that aerobic exercise is the most effective training modality, and low-to-moderate intensity and mixed intensity are better compared with high intensity to decrease NLRP3 inflammasome activation-related inflammatory cytokine IL-1β and IL-18 (Ding and Xu, 2021).

One more mechanism through which exercise promotes an anti-inflammatory response is a downregulation of pro-inflammatory toll-like receptors (TLRs) (Abbasi et al., 2014). Aerobic exercise reduces the expression/activation of TLR4 on the surface of monocytes (Lancaster et al., 2005). Since these monocytes are the predecessors of tissue macrophages, the exercise-induced decline in monocyte TLR4 expression is probably a key mechanism to the anti-inflammatory responses of exercise (Collao et al., 2020). Exercise led to the downregulation of TLR4 (Quiroga et al., 2020). The interaction between the microbiota and the TLRs and NLRP3 inflammasome activation, resulting in low-grade inflammation, is well-established (Quiroga et al., 2020).

Physical exercise activates peroxisome proliferator–activated receptor coactivator 1 alpha (PGC-1α) pathway, a signaling factor that is associated with decreasing pathological myocardial remodeling, lowering blood pressure and decreased deposition of collagen (Whitehead et al., 2018), as well as reducing systemic inflammatory profile by preventing the infiltration of macrophages, TNF-α, and inducible nitric oxide synthase (iNOS), involving inhibition of cytokines and pro-inflammatory factors in the blood (Botta et al., 2013). PGC-1α results in the elevated expression of FNDC5, which release Irisin (Reza et al., 2017).

**FIGURE 2:**
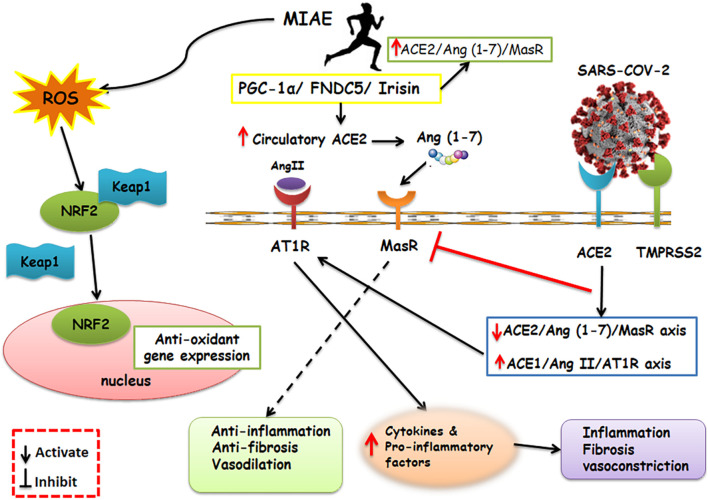
Schematic representation of the main molecular pathways activated by SARS-CoV-2 and aerobic physical exercise and their respective consequences. On the right of the panel, the SARS-CoV-2 is shown, which relies upon ACE2 and TMPRSS2 to enter the host cells. SARS-CoV-2 upregulates the expression of ACE1/Ang-II/AT1R and inhibits the MasR which results in exacerbating inflammatory response. Contrarily, a shifted RAS balance toward the ACE2/Ang 1–7/Mas receptor axis after aerobic physical exercise was a reason for defensive outcomes. Furthermore, exercise activates PGC-1α-FNDC5/Irisin pathway. Irisin play an important role in anti-inflammatory pathways including Nrf2. Nrf2 results in transformation of cells subject to oxidative stress. In normal circumstances, Nrf2 is placed in the cytoplasm bound to its Keap1, which is its inhibitor. When ROS caused by exercise is available, the Keap1 and Nrf2 connection detaches and Nrf2 moves to the nucleus, where it arouses the antioxidant and anti-inflammatory activity. MIAE, Moderate intensity aerobic exercise; ACE2, Angiotensin converting enzyme2; Ang II, Angiotensin II; Ang 1–7, Angiotensin 1–7; AT1R, Angiotensin II receptor type 1; MAS R, Mas receptor; ROS, Reactive Oxygen Species; FNDC5, fibronectin type III domain-containing protein 5; PGC-1α, Peroxisome proliferator-activated receptor gamma coactivator 1-alpha; TMPRSS2, Transmembrane serine protease 2; Nrf2, nuclear factor erythroid 2–related factor 2; KEAP1, Kelch-like ECH-associated protein 1.

Also, by accumulation of neutrophils, the creation of ROS and pro-inflammatory molecules raises (Yan and Spaulding, 2020), which are associated with acute lung injury, the most common cause of morbidity and mortality in COVID-19 (Nobari et al., 2021b). Mitochondria is a place for ROS production and exercise is a potent mediator of ROS (Nobari et al., 2021a). Irisin is a skeletal muscle myokine (Nobari et al., 2021a), which could play a role in anti-oxidative pathways involving the transcription factor nuclear factor erythroid 2-related factor 2 (Nrf2) (Wang et al., 2016), and even on the expression of many distinct genes related to SARS-CoV-2 infection (de Oliveira et al., 2020). Irisin has a positive correlation with improved lung function (Nobari et al., 2021b). Furthermore, Nrf2 results in the transformation of cells subject to oxidative stresses (Ruhee and Suzuki, 2020). In normal circumstances, Nrf2 is placed in the cytoplasm bound to its Keap1, which is its inhibitor, and leads to consequent degradation of Nrf2. When ROS is available, the connection between Kelch like ECH associated protein 1 (Keap1) and Nrf2 detaches and Nrf2 moves to the nucleus, where it arouses the antioxidant feedback (Ruhee and Suzuki, 2020). Exercise causes Nrf2 migration to the nucleus, and this arouses antioxidant and anti-inflammatory activity (Wang et al., 2016; Figure 2).

Considering that the universal concern about chronic diseases is constantly increasing, the advancement of approaches to a life-long rise in regular physical activity at the social level is required (Alack et al., 2019). Generally, the direct and indirect anti-inflammatory effects of moderate intensity aerobic exercise cause a great percentage of its preventive and therapeutic capacity. The fact that exercise intervention is able to induce a fundamental change in downstreaming immune responses could be used to develop concepts that could influence the development and treatment success of infectious diseases.

## Returning to Exercise Training After COVID-19 Recovery

Many recovered patients with COVID-19, notably those manifesting severe symptoms during the infection phase, are not able to improve functional life, a sign of returning to the normal life after being discharged (Giallauria et al., 2018). Subsequently, slow and gradual return to the earlier level and at the same time being cautious about any clinical or cardiovascular symptoms have been indicated (Salman et al., 2021). Once the person has recovered from COVID-19, safety-focused physical activity approaches should be considered to reduce the likelihood of risks. After recovering from COVID-19, physical and psychological situation of the individual play a key role for returning to activity (Salman et al., 2021).

A week from COVID-19 symptom onset, generally a decline in severe infection occurs (Salman et al., 2021). Accordingly, a return to PE is only allowed following at least 7 days symptom-free period (Barker-Davies et al., 2020).

In a study on performance athletes who had mild-to-moderate illness, it was recommended that anaerobic sports like golf can be performed earlier but before restarting aerobic exercise, daily routine activities should be obtained comfortably and a 500 m walk on the flat surface should be easily achievable without feeling extra fatigue or difficulty in breathing (Elliott et al., 2020). Nonetheless, as some people may not be able to walk 500?m without shortness of breath in the normal situation, dealing with the individual’s pre-COVID-19 baseline ability has been suggested (Salman et al., 2021).

The people who performed recreational exercise activities for general physical fitness preillness, after recovering from COVID-19 with mild-to-moderate manifestations, without hospitalization and concerning heart-related symptoms should be able to proceed moderate intensity recreational exercise according to physical activity guidelines for Americans (Calabrese and Neiman, 2021; Salman et al., 2021), which recommends changing sedentary behaviors by moving more and sitting less, getting at least 150 min of moderate aerobic activity or 75 min of vigorous aerobic activity per week and performing exercises that develop muscular strength on two or more days a week (Piercy et al., 2018).

Generally, recovery from respiratory viral infections takes 2–3 weeks, which is related to the time it takes your immune system to develop cytotoxic T cells crucial to clear the virus from infected cells. After this period, when symptoms are over, it is safe to start exercising on a routine basis, but it is suggested to start slowly (Zhu, 2020). However, definite recommendations are not available at present.

## Exercise Training in Those With Active COVID-19

During the acute phase of infection, exercise may cause increased viral replication, an intense pro-inflammatory response ending in heightened cellular necrosis (Kiel et al., 1989). After COVID-19 disease, due to the spread of infection, the patients will not be able to exercise (Zhu et al., 2020; Nobari et al., 2021a). Hence, it was recommended to cease exercise in case of active COVID-19 infection and the presence of any of the following symptoms: severe sore throat, body aches, shortness of breath, general fatigue, chest cough, or fever (Zhu, 2020). Clinical features of COVID-19 have been classified into: mild [no dyspnea, no low blood oxygen saturation (SatO2)], moderate (dyspnea, SatO2 94 to 98%, radiological signs of pneumonia), severe (dyspnea, SatO2 ≤ 93%, respiratory rate (RR) > 30/min, radiological progression of lesions, with O2 supplementation required, eventually with non-invasive ventilation), and critical (patients need mechanical ventilation) (Carda et al., 2020). According to several reports, the lungs are exposed to the virus in severe patients with COVID-19, so these individuals are completely unable to exercise; however, in patients with mild disease, short-term moderate exercises can be a good solution (Zhu, 2020).

A myocardial injury due to COVID-19 has been proposed, which is a combination of early acute phase of viral induced myocarditis and a sub-acute pro-inflammatory response leading to cardiac damage (Siripanthong et al., 2020). Over the chronic phase, clinical manifestations of SARS-CoV-2 myocarditis range from relatively mild symptoms, like fatigue and dyspnea (Inciardi et al., 2020), chest pain, or chest tightness on exertion (Zeng et al., 2020) to heart failure (Inciardi et al., 2020). In case of heart manifestations (palpitations, syncope, chest pain, dyspnea, unexplained increase in heart rate) after COVID-19 infection, avoid moderate to high intensity exercise (McKinney et al., 2021). About 7–20% of sudden cardiac deaths (SCD) in young athletes are due to myocarditis (Maron et al., 2016). So, a more cautious approach should be applied when returning to exercise after myocarditis. During COVID-19 with myocarditis and even 3–6 months after recovery, strenuous activity and exercise should be restricted (McKinney et al., 2021).

Besides, COVID-19, which is initially a respiratory disease, has the potency to convert into a motor impairment after acute stages, associated with the length of time the patient spends in intensive care (Piquet et al., 2021). Many COVID-19 patients experience serious motor loss (Hekmatikar et al., 2021), severe fatigue, and deficiency in the legs and arms muscles (Islam et al., 2020), so they may suffer from muscle atrophy and physical impairment, which leads to limited movement and loss of muscle function. In this context, exercise could potentially play a key role during recovery from COVID-19.

Inpatient rehabilitation for patients with COVID-19 was associated with significant functional and respiratory enhancement, especially in severe cases (Piquet et al., 2021). Also, a recent study proposed that rehabilitation and breathing exercises in patients are effective in preventing muscle atrophy or at least its rapid progression (Nobari et al., 2021a). A systematic review with meta-analyses showed that aerobic training combined with breathing exercises led to significant advancement in exercise capacity, dyspnea, and quality of life in patients with idiopathic pulmonary fibrosis (Hanada et al., 2020).

Nevertheless, because of respiratory tract infection, achieving aerobic exercises for patients with COVID-19 would be exceedingly tough. Accordingly, skeletal muscle exercise with minimum lung involvement such as isometric training (muscle contraction with muscle-tendon unit at a constant length), which includes 5–10 repetitions of 5 s per contraction, 5 days per week should be preferred (Khoramipour et al., 2021; Nobari et al., 2021a).

Besides, as lung is the eventual organ affected by SARS-COV-2, it is vital to target exercise approaches on cardiorespiratory muscle training to enhance respiratory function (Rothan and Byrareddy, 2020). Respiratory muscles like other muscles respond to stimulation (Suzuki, 2019). Rising respiratory load is investigated as the primary stimulation for these muscles and can cause developed strength and endurance (Vargas-Mendoza et al., 2019). Respiratory muscle training (RMT) is an applicable method to enhance respiratory and exercise performance, which incorporates (1) decreased respiratory muscle fatigue, (2) delayed respiratory muscle metaboreflex activation, and (3) improved SaO2 preservation and blood supply to active muscles (Álvarez-Herms et al., 2019), all of which are exactly desirable outcomes for patients with COVID-19. A 2-week RMT promoted pulmonary function, dyspnea, and functional performance in recovered ICU patients with COVID-19 after ensuring removing mechanical ventilation (Abodonya et al., 2021). So it was recommended to include RMT program in the COVID-19 management approaches, especially in ICU patients (Abodonya et al., 2021).

## Conclusion

Aerobic moderate intensity PE shows the potential as a novel non-pharmacological intervention against COVID-19 pandemic. In the context of previously reported studies, we hypothesized that the physiological responses associated with aerobic exercise elicit effects that could stimulate the ACE2-Ang-(1–7)-Mas receptor axis and anti-inflammatory responses, which act in favor of the immune system and even promote potent effects against infectious diseases like COVID-19. Whether aerobic exercise increases ACE2 expression and whether it adjusts susceptibility to SARS-CoV-2 and the severity of COVID-19 disease should be considered in large groups. Along with scientific evidence provided here, healthy individuals will take advantage from aerobic moderate intensity exercise and will improve immune function, which will be a relevant asset in struggle against COVID-19.

Further experimental and clinical researches of the definite role of aerobic PE and its rule in RAS system and anti-inflammatory process are necessary to address its probable advantage and beneficial effect on prevention against COVID-19. However, the available evidence strengthens the recommendation for moderate intensity PE as an effective strategy during the pandemic period for various reasons, namely, lowering the metabolic and cardiovascular risk factors. In this way, the aerobic moderate intensity exercise prescription would diminish future confusion about this disease, and should be highly advocated in healthy individuals.

## Data Availability Statement

The original contributions presented in the study are included in the article/supplementary material, further inquiries can be directed to the corresponding author/s.

## Author Contributions

HA and AF conceptualized the perspective idea. AF prepared first draft of the manuscript. HA and KS revised the text of the manuscript. All authors read and approved the final version of the manuscript.

## Conflict of Interest

The authors declare that the research was conducted in the absence of any commercial or financial relationships that could be construed as a potential conflict of interest.

## Publisher’s Note

All claims expressed in this article are solely those of the authors and do not necessarily represent those of their affiliated organizations, or those of the publisher, the editors and the reviewers. Any product that may be evaluated in this article, or claim that may be made by its manufacturer, is not guaranteed or endorsed by the publisher.
